# VP2 mediates the release of the feline calicivirus RNA genome by puncturing the endosome membrane of infected cells

**DOI:** 10.1128/jvi.00350-24

**Published:** 2024-04-09

**Authors:** Weiyao Sun, Ming Wang, Zhibin Shi, Pengfei Wang, Jinhui Wang, Bingchen Du, Shida Wang, Zhenzhao Sun, Zaisi Liu, Lili Wei, Decheng Yang, Xijun He, Jingfei Wang

**Affiliations:** 1 State Key Laboratory for Animal Disease Control and Prevention, Harbin Veterinary Research Institute, Chinese Academy of Agricultural Sciences, Harbin, China; University of Michigan Medical School, Ann Arbor, Michigan, USA

**Keywords:** genome release, VP2 protein, calicivirus, membrane penetration

## Abstract

Feline calicivirus (FCV) is one of the few members of the *Caliciviridae* family that grows well in cell lines and, therefore, serves as a surrogate to study the biology of other viruses in the family. Conley et al. (14) demonstrated that upon the receptor engagement to the capsid, FCV VP2 forms a portal-like assembly, which might provide a channel for RNA release. However, the process of calicivirus RNA release is not yet fully understood. Our findings suggest that the separation of the FCV capsid from its genome RNA (gRNA) occurs rapidly in the early endosomes of infected cells. Using a liposome model decorated with the FCV cell receptor fJAM-A, we demonstrate that FCV releases its gRNA into the liposomes by penetrating membranes under low pH conditions. Furthermore, we found that VP2, which is rich in hydrophobic residues at its N-terminus, functions as the pore-forming protein. When we substituted the VP2 N-terminal hydrophobic residues, the gRNA release efficacy of the FCV mutants decreased. In conclusion, our results suggest that in the acidic environment of early endosomes, FCV VP2 functions as the pore-forming protein to mediate gRNA release into the cytoplasm of infected cells. This provides insight into the mechanism of calicivirus genome release.

**IMPORTANCE:**

Research on the biology and pathogenicity of certain caliciviruses, such as Norovirus and Sapovirus, is hindered by the lack of easy-to-use cell culture system. Feline calicivirus (FCV), which grows effectively in cell lines, is used as a substitute. At present, there is limited understanding of the genome release mechanism in caliciviruses. Our findings suggest that FCV uses VP2 to pierce the endosome membrane for genome release and provide new insights into the calicivirus gRNA release mechanism.

## INTRODUCTION

Caliciviruses are single-stranded RNA viruses belonging to the *Caliciviridae* family, which consists of 11 genera. Seven of these genera, *Sapovirus, Norovirus*, *Nebovirus*, *Lagovirus*, *Recovirus*, *Valovirus,* and *Vesivirus,* infect mammals and cause various diseases (1, 2). Notably, members of the *Sapovirus* and *Norovirus* genera are important human pathogens and cause outbreaks of acute gastroenteritis, especially in children (3, 4). The study of the biology and pathogenesis of these human caliciviruses is hindered by the lack of easy-to-use cell culture system (5, 6). Feline Calicivirus (FCV) is a primary pathogen causing pneumonia or severe upper respiratory tract disease in cats and serves as an excellent model for studying the biology of caliciviruses due to its ability to replicate in cell lines (7, 8).

The capsid of caliciviruses has a *T* = 3 icosahedral symmetry structure. It is composed of 180 copies of the major capsid protein (VP1) and 12 copies of the minor capsid protein (VP2) (9, 10). To initiate infection, FCV binds to the feline tight-junction cell-adhesion molecule A (fJAM-A). It then enters the host cells through clathrin-mediated endocytosis (11, 12). A previous study indicated that FCV VP2 is crucial for producing infectious virions (13). More recently, the cryo-EM structure of FCV (F9 strain) complexed with fJAM-A revealed that the receptor’s engagement with VP1 induces VP2 to form a portal-like assembly. This assembly is thought to act as a channel for genome RNA release during infection (14).

During a viral infection, the release of the viral genome into a host cell is a crucial step. Unlike enveloped viruses, such as influenza A viruses and Coronaviruses which deliver their genomes into the cytoplasm through membrane fusion (15, 16), nonenveloped viruses use a variety of mechanisms to disrupt host membranes for genome release (17, 18). Reoviruses accomplish genome release by disrupting the endosomal membrane, delivering the viral sub-infectious particles into the cytoplasm to initiate viral genome replication (19, 20). Poliovirus and enteroviruses, on the other hand, translocate their genomes through intact membranes using channels formed by membrane-associated capsid proteins (21–24). Caliciviruses are also naked viruses, but their genome RNA release process has not been clearly determined.

In this study, we use a combination of cell assay with fluorescence-labeled viruses and a cell-free assay employing receptor-decorated liposomes to investigate the FCV gRNA release. We found that FCV VP2 serves as the pore-making protein, mediating viral gRNA release in the early endosomes of infected cells. These findings offer new sights into the calicivirus gRNA release mechanisms and are valuable for the development of antiviral drugs.

## RESULTS

### FCV RNA release in early endosome of infected CRFK cells

**Fig 1:**
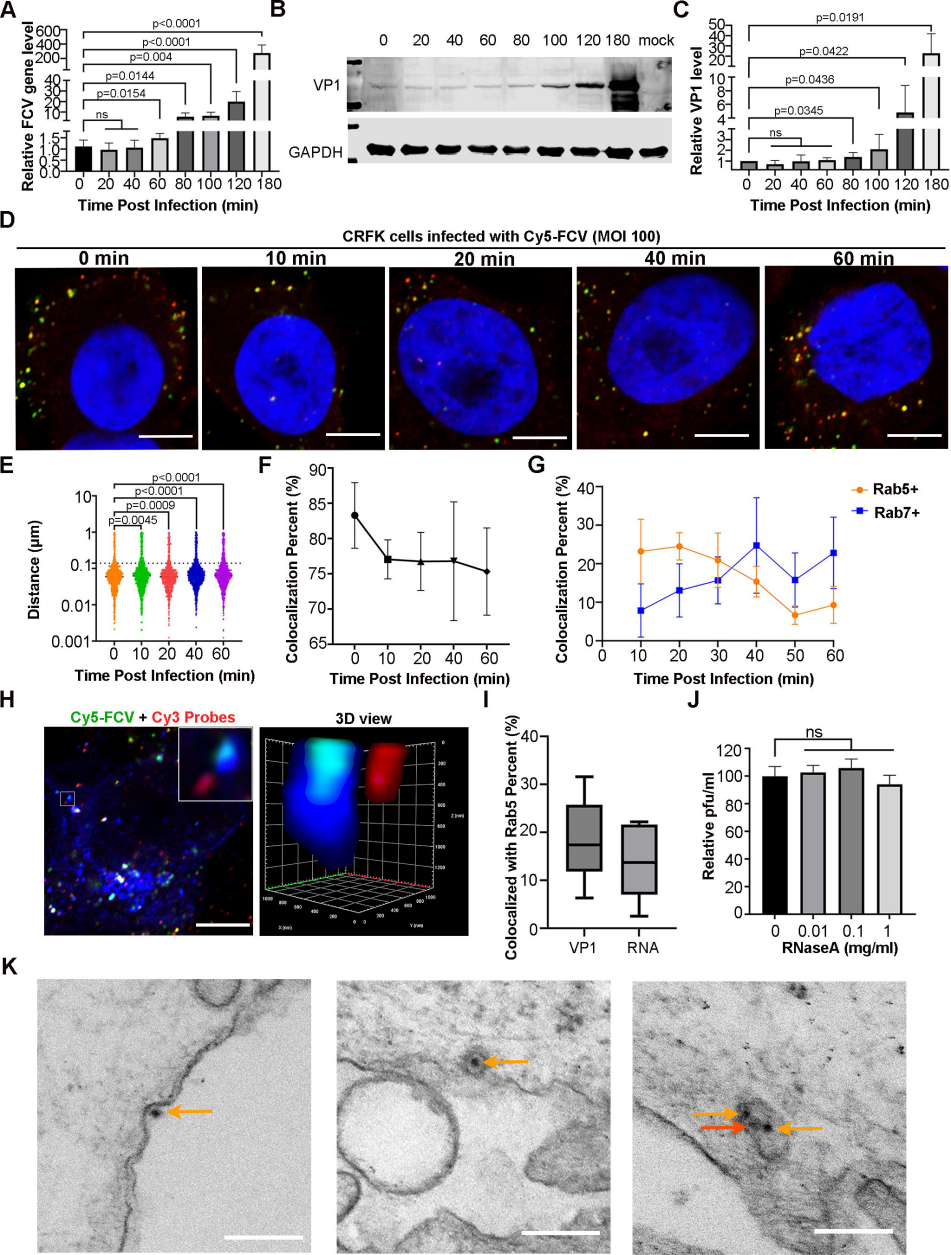
FCV RNA release in the early endosome. (**A**) Quantification of the viral RNA levels at the indicated time points post infection. After FCV (100 MOI) adsorption for 60 min at 4°C, cells were transitioned to 37°C and viral RNA was extracted and tested using a RT-qPCR. (**B**) WB results of VP1 levels. FCV infection was performed as described in (**A**), and VP1 was detected using a mAb against VP1 (Abcam). (**C**) Quantitative presentation of VP1 levels is shown in (**B**). The data in (**A and C**) come from three independent experiments. The Student’s *t*-test was used to measure the difference between two independent groups, and the *P*-value for each comparison is provided. (**D**) The confocal microscope images show the colocalization of FCV capsids (indicated by green fluorescence signals) and gRNA (indicated by red fluorescence signals). The nucleus was stained with DAPI Scale bar, 5 µm. (**E**) The scatter plot depicts the variation in the distance between the viral capsid and RNA. The statistical analysis was conducted using the Student’s *t*-test with more than 1,000 pairs of spots. The *P* value is labeled for each comparison. (**F**) The curve plots show the change in the percentage of colocalization between viral RNA and the capsid. (**G**) Quantify of the colocalization percent of VP1 with either Rab5 or Rab7 vesicles at the indicated times. (**H**) The confocal microscope images show colocalization of viral RNA, capsids, and early endosomes. The inset (left) and 3D view (right) show the separation of viral RNA from the capsids within the early endosomes. Scale bar, 5 µm. (**I**) Quantify of percentage of VP1 and RNA colocalized with the early endosomes at 10 min post infection. (**J**) Bar chart shows the relative plaque number of FCV in the presence of 0–1 mg/mL RNase A. Relative pfu/mL were expressed as percentage of no RNase A control. The statistical analysis was source from three independent experiments and calculated by the Student’s *t*-test. (**K**) Representative TEM images of FCV-infected cells. The images display FCVs attached to the cell surface (left), within an endosome through clathrin-mediated endocytosis (middle), and inside an endosome via macropinocytosis (right). The brown and red arrows point to the intact and a likely empty FCV particles. Scale bar, 200 nm.

We examined the timing of FCV RNA release in the infected cell. FCV was incubated with Crandell Reese feline kidney (CRFK) cells at 4°C for 1 h, allowing for attachment, before moving the cells to 37°C to initiate virus entry. Viral RNA and VP1 levels during infection were detected by real-time quantitative PCR (RT-qPCR) and Western blot (WB), respectively. The results revealed a significant increase in viral RNA and VP1 levels at 60 and 80 min post-attachment (Fig. 1A through C), suggesting that the majority of FCV RNA released within 60 min post-attachment. To more precisely determine the timing and location of viral RNA release, we prepared Cy5-labeled FCVs (Fig. S1A through C) and developed a smiFISH with Cy3-labeled probes targeting viral RNA (Fig. S2). We then conducted confocal microscope analyses on the FCV-infected cells. The separation of viral RNA and capsid was determined by measuring the distance between the closest red and green fluorescence signals. We found a rapid separation of viral RNA and capsid occurred within 10 min post-absorption (Fig. 1D through F). During this period, we analyzed the colocalization of VP1 with the early endosomes (indicated by eGFP-rab5) and late endosomes (indicated by eGFP-rab7). As expected, VP1 was located dominantly within the early endosome at 10 min post-attachment (Fig. 1G). We also investigated the colocalization among VP1, viral RNA, and Rab5 (indicating early endosomes) at 10 min post-attachment. We found the separation of viral RNA from the capsid and the early endosomes (Fig. 1H). Consistently, the colocalization percentage of VP1 with Rab5 was higher than that of RNA with Rab5 (Fig. 1I), and the RNA protection assay revealed that the presence of RNase A in the endosomes did not affect the viral titers (Fig. 1J), indicating that FCVs in the early endosomes have released their RNA into the cytoplasm. Furthermore, an EM examination of FCV-infected cells at 10 min post-attachment revealed that a few virions were attached to the cell surface. Most of the viruses, however, had entered the cell through either clathrin-mediated endocytosis or macropinocytosis. Both intact and empty FCV particles were observed in the endosomes (Fig. 1K). Together, these findings suggest that FCV likely releases its gRNA into the cytoplasm by piercing the endosome membranes.

### Establishment of an *in vitro* FCV RNA release model

**Fig 2:**
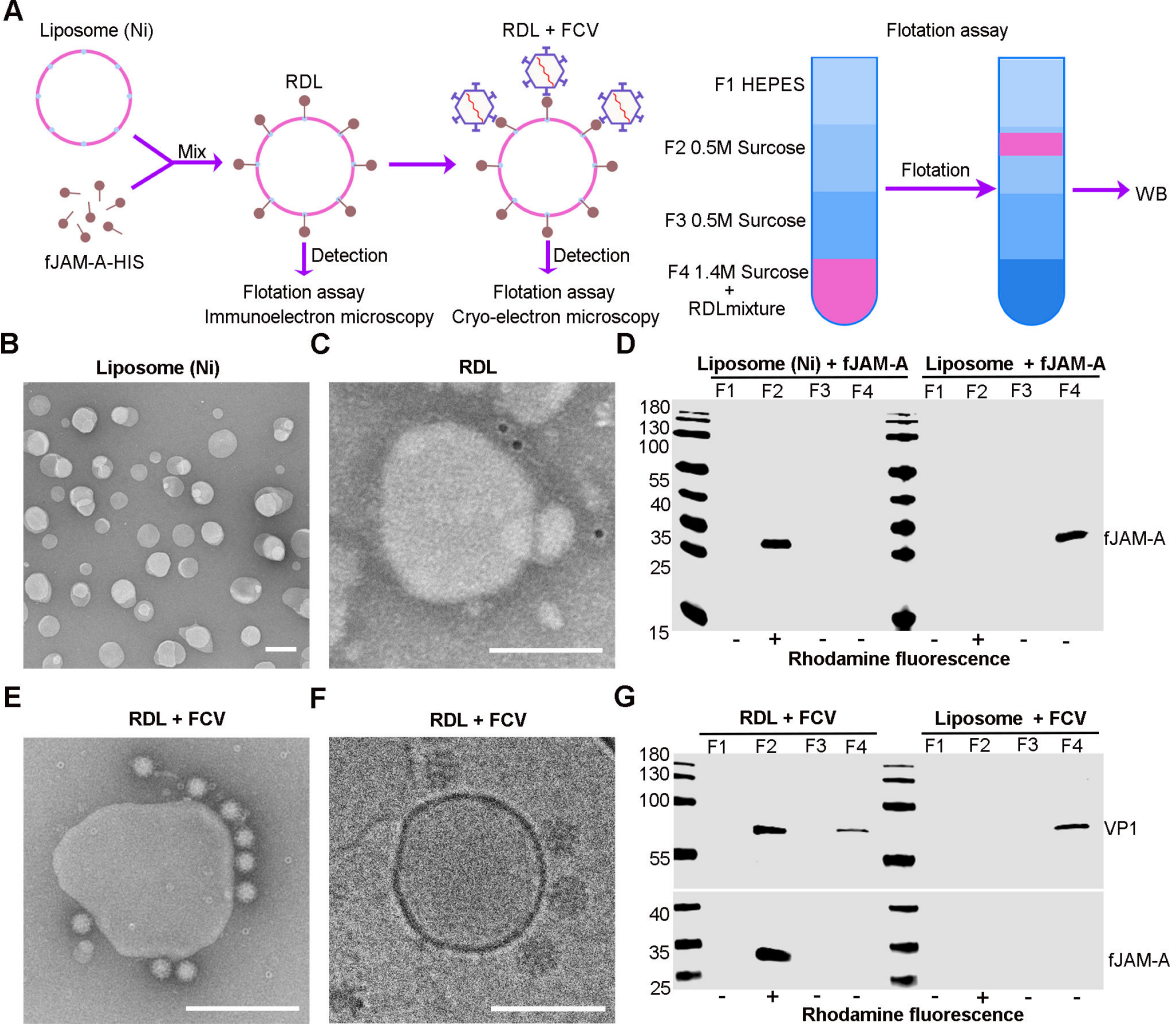
Preparation of fJAM-A-decorated liposomes. (**A**) Schematic diagram of the preparation of receptor-decorated liposomes (RDL) and detection methods. (**B**) TEM image showing the morphology of nickel-charged liposomes. Scale bar = 200 nm. (**C**) Immunoelectron microscopy image showing the attachment of fJAM-A on the nickel-charged liposomes. Scale bar, 200 nm. (**D**) WB result of the binding of fJAM-A with liposomes. After flotation assay, fJAM-A was detected by anti-His antibody (GeneTex) and in the same F2 layer with liposomes. (**E and F**) FCV attachment on the RDL surface was examined using TEM. Scale bar, 200 nm (**E**), and Cryo-EM, scale bar,100 nm (**F**). (**G**) WB result of the attachment of FCV on RDL. After flotation assay, FCV was detected by anti-VP1 antibody (Abcam) in the same F2 layer with liposomes and fJAM-A.

To test our hypothesis, we built a receptor-decorated liposomes (RDL) model to replicate the FCV RNA release process in endosomes *in vitro*. Initially, we prepared nickel-charged liposomes as reported earlier (25) and then incubated them with the fJAM-A-His (extracellular domain of fJAM-A linked with His tag) to produce RDL. Using transmission electron microscopy, flotation assays, and WB, we confirmed the binding of fJAM-A-His to liposomes and FCV to RDLs (Fig. 2A). The liposomes were spherical vesicles with an average diameter of about 100 nm (Fig. 2B). The fJAM-A-His attached on the liposome surface was specifically detected by immune gold labeling (Fig. 2C) and found in the same gradient fraction of liposomes by flotation and subsequent WB assays (Fig. 2D). We then validated the FCV-binding capability of RDLs using negative staining (Fig. 2E), cryo-EM analysis (Fig. 2F), and flotation assays (Fig. 2G). The results consistently showed that the RDLs could bind FCV with high efficacy.

### FCV RNA release through pore formation in the membrane

**Fig 3:**
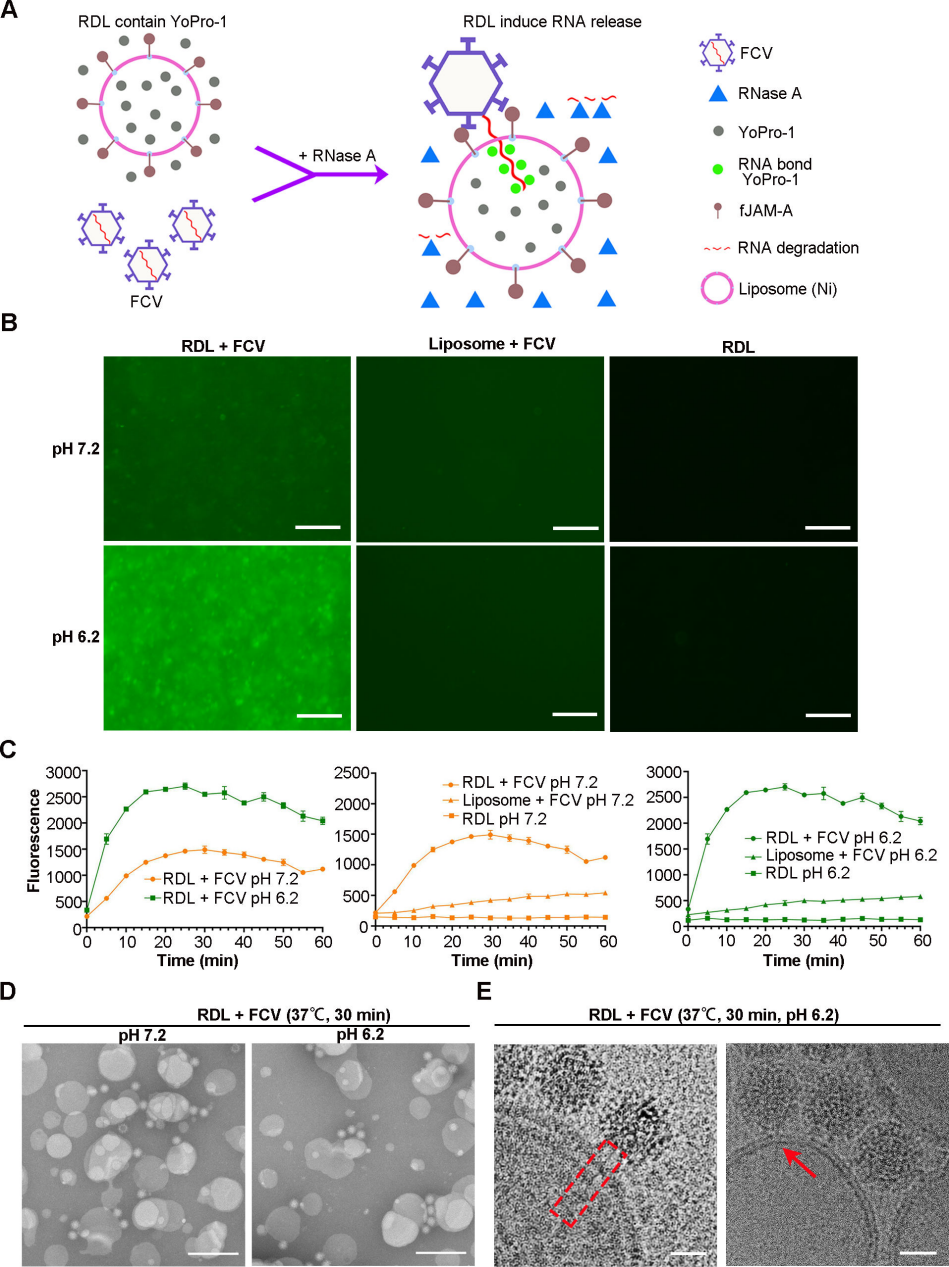
FCV RNA release through membrane penetration. (**A**) The schematic illustration of the FCV RNA release model. (**B**) Fluorescence microscopy images of mixtures incubated at pH 7.2 (top) and pH 6.2 (bottom) for 30 min. Bars, 100 µm. (**C**) Comparison of the fluorescence intensity of the mixtures at different pH conditions. A PHERAstar FS plate reader was used to record at a 5-min interval for 60 min at 37°C. The data in the above statistical analysis are sourced from three independent experiments. (**D**) TEM images show the integrity of FCV attached on the surface of RDL at various pH conditions. Bars, 200 nm. (**E**) Cryo-EM image showing a likely RNA release process. In the red dotted rectangle (left), showing a linear RNA-like element across the membrane of a liposome. The red arrow (right) point to an RNA-like element passing through a pore in the liposome membrane. Bar, 25 nm.

To visualize the FCV RNA release process, we modified our RDL model by adding Yopro-1, a substance that enhances fluorescence upon nucleic acid binding, and RNase A to digest RNAs outside the liposomes (Fig. S3A and B). The fundamental concept of this model is that detectable fluorescence signals stem solely from RNA binding with YoPro-1 inside the liposome (Fig. 3A). We used this model to study FCV RNA release under pH conditions of 7.2 and 6.2. We found that fluorescence signals in FCV-bound RDLs at pH 6.2 were much stronger than those at pH 7.2 and receptor-free controls 30 min after incubation (Fig. 3B). We monitored the change in fluorescence intensity in all groups for 60 min using a PHERAstar FS plate reader. Fluorescence intensity increased rapidly in the FCV-bound RDL group at both pH levels within the first 20 min of incubation. Notably, the signal intensity at all time points in the pH 6.2 group was significantly higher than in the pH 7.2 group, suggesting acidic conditions promote the viral nucleic acid release (Fig. 3C). TEM examination revealed that the viral capsids remained intact and closely attached to the liposome surfaces under all pH conditions (Fig. 3D). Furthermore, cryo-EM analysis of the RDL-FCV complex at pH 6.2 revealed a presumed linear RNA element across the double membrane (Fig. 3E). In summary, these results indicate that FCV releases its gRNA into the liposomes by forming a pore in the membrane, and this process is facilitated by acidic conditions.

### VP2 serves as a membrane binding and penetration peptide

**Fig 4:**
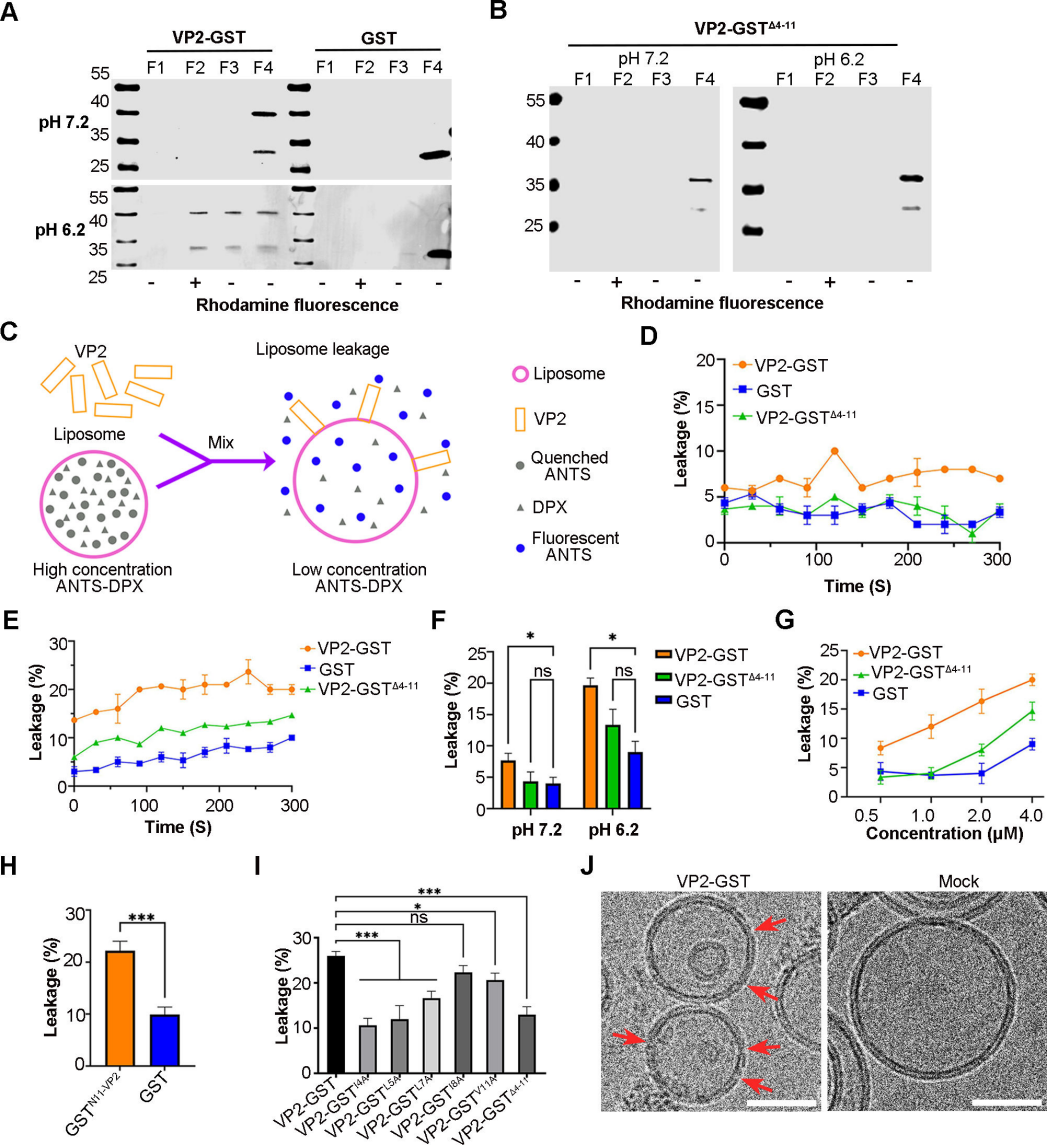
VP2 binds with liposomes and induces membrane permeability in low pH through N-terminal. (**A and B**) WB results of flotation assay of VP2-GST (**A**) and VP2-GST^Δ4-11^ (**B**) at pH 7.2 and 6.2 conditions. The liposomes were mixed with the proteins for 30 min at RT. The mixture was submitted to a flotation assay, and then, the fractions were then analyzed through WB by using anti-GST antibody (GeneTex). (**C**) The schematic illustration of the leakage assay. (**D and E**) Comparison of liposome leakage rates led by the proteins at pH 7.2 (**D**) and pH 6.2 (**E**). (**F**) Bar chart shows the difference of the liposome leakage rate caused by the VP2-GST at pH 7.2 and pH 6.2 conditions. (**G**) Comparison of liposome leakage rates led by different concentrations of the proteins at pH 6.2. (**H**) Bar chart shows the leakage of liposome caused by GST^N11-VP2^ at pH 6.2. Error bars represent SD of the mean from three independent experiments. Asterisks indicate statistical significance calculated by the Student’s *t*-test, (***P < 0.001). (**I**) Bar chart shows liposome leakage caused by mutant VP2 proteins at pH 6.2. Error bars in F and I represent the SD from three independent experiments. Asterisks indicate statistical significance among proteins calculated by the one-way analysis of variance (ANOVA) (*P < 0.05, ***P < 0.001). (**J**) Cryo-EM images illustrate liposome morphology after interaction with VP2. The red arrows (left) point to the pores in the liposome membrane induced by VP2, while the right panel shows an intact liposome without the addition of VP2. Bar, 50 nm.

A previous study indicated that FCV VP2 forms a portal-like assembly once fJAM-A engages with VP1. This suggests that VP2 could potentially mediate the RNA release of FCV. We performed hydrophobicity and amino acid sequence analyses on VP2 and discovered that the residues at its N-terminus (aa 4–11) were highly hydrophobic and conserved among FCV strains. Consequently, we hypothesized VP2 might serve as the pore-making protein to mediate the gRNA release and its N-terminus could bind and penetrate double-layer membranes. To test this assumption, we prepared the GST-tagged VP2 and VP2-GST^Δ4-11^ proteins (Fig. S4A and B) and evaluated their liposome binding and penetration properties. Flotation assays revealed that VP2-GST was associated with the liposomes at pH 6.2, whereas VP2-GST^Δ4-11^ did not bind to liposomes at either pH 6.2 or pH 7.2 (Fig. 4A and B). We conducted immune-gold labeling experiments to test the binding of VP2-GST and VP2-GST^Δ4-11^ to liposomes at pH 6.2. The results showed that VP2-GST specifically binds to the surface of liposomes, whereas VP2-GST^Δ4-11^ lost this ability (Fig. S4C). We then designed a liposome leakage assay to test the membrane penetration property of VP2. High concentrations of ANTS and DPX were encapsulated in the liposomes, and upon leakage, ANTS fluorescence was no longer quenched by DPX and was detectible under a fluorescence microscope (Fig. 4C). This assay was initially verified by adding 1% TritonX-100, leading to a significant enhancement in fluorescence signal, regardless of pH conditions (Fig. S4D). The leakage assays indicated that VP2-GST incubation resulted in higher liposome leakage at both pH 6.2 and pH7.2 compared to VP2-GST^Δ4-11^ and GST (Fig. 4D through F). We also demonstrated that the liposome leakage caused by VP2-GST was dose-dependent (Fig. 4G). Moreover, GST^N11-VP2^, in which GST was fused with the N-terminal 11 amino acids of VP2, induced a higher level of liposome leakage compared to GST alone, suggesting that the N-terminus of VP2 has a membrane permeability capacity (Fig. 4H). To further assess the impact of hydrophobic amino acids in the N-terminus of VP2 on its membrane permeability, we prepared the GST-tagged mutant VP2 proteins and used them to conduct liposome leakage assays (Fig. S4A and B). The results showed that all the mutant proteins except I8A caused a lower liposome leakage compared to VP2-GST (Fig. 4I). We further investigated the morphological changes in liposomes following their interaction with VP2 at pH 6.2 using cryo-EM and observed the formation of pores, less than 10 nm in size, in the liposome membrane (Fig. 4J). These findings suggest that VP2 creates pores in the membrane through its N-terminus, leading to liposome leakage.

### The substitution of hydrophobic residues with alanine at the N-terminus of VP2 decreases the efficiency of FCV RNA release

**Fig 5:**
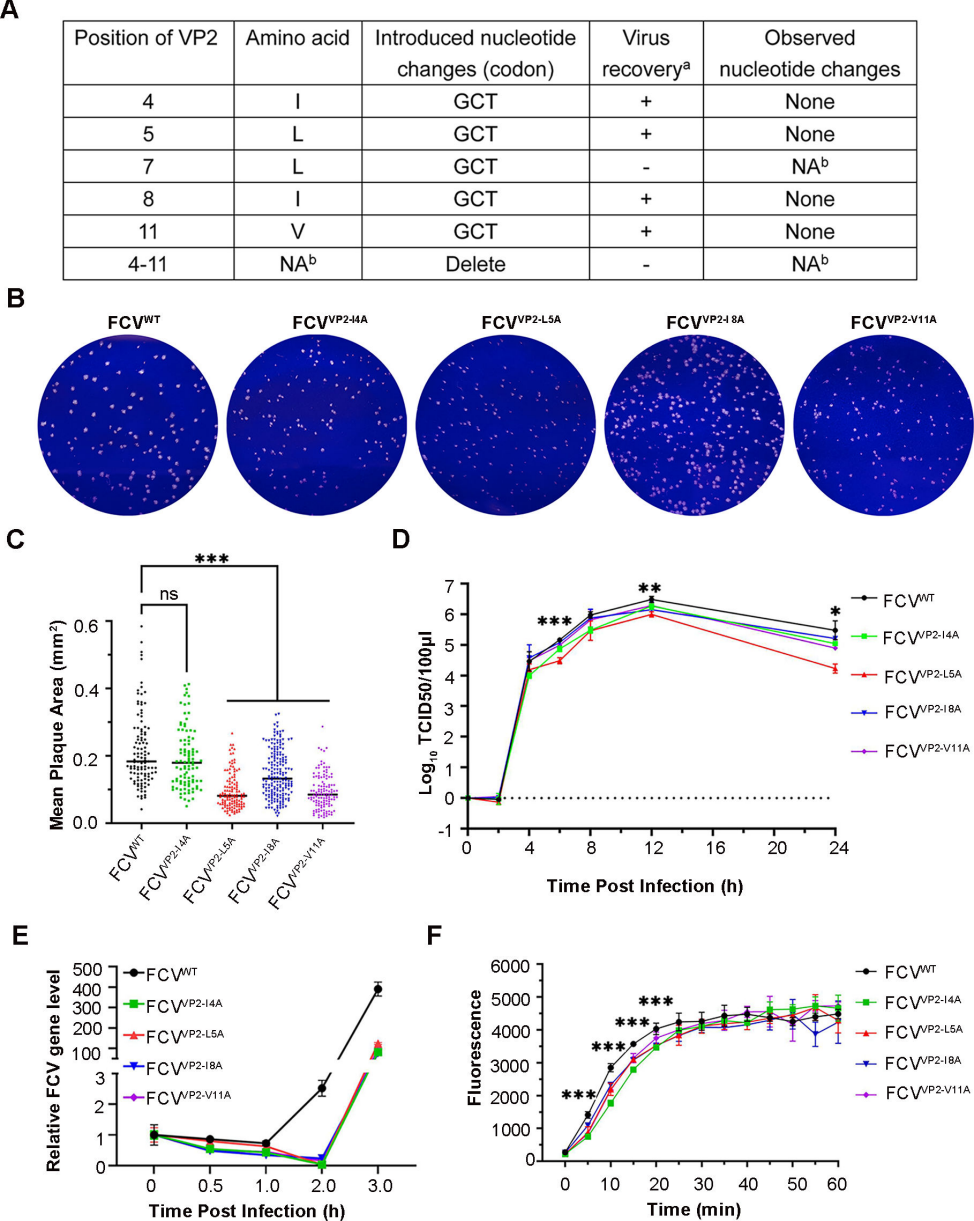
Alanine substitutions of VP2 N terminus decrease FCV RNA release. (**A**) Mutant viruses rescue results. ^a^ +, clones that yielded viable virus progeny; −, clones that did not yield viable virus progeny; ^b^ NA, not applicable. (**B**) Plaque phenotypes of FCV^WT^ and mutant strains. (**C**) Comparison of plaque areas between FCV^WT^ and the mutant strains. The plaque area was analyzed using Image J (version 1.8.0). The average plaque area for each strain is represented with a black line. Statistical significance between area of mutant stains and that of FCV^WT^ was calculated by one-way ANOVA (***P < 0.001). (**D**) One step growth curve shows the different growth property of FCV^WT^ and mutant strains. Error bars represent the SD from three independent experiments, and asterisks indicate statistical significance among FCV strains calculated by one-way ANOVA (*P < 0.05, **P < 0.01, ***P < 0.001). (**E**) Line plots show the viral RNA fold differences among the viruses. For quantification, normalized viral mRNA was expressed as a percentage of the amount at 0 mpi. (**F**) Comparison of the fluorescence intensity among the mutant viruses and FCV^WT^ at pH 6.2. FCV^WT^ or mutant viruses mixed with RDL and the fluorescent signals were recorded using a PHERAstar FS plate reader. Data from three independent experiments and asterisks indicate statistical significance among FCV strains calculated by one-way ANOVA (***P < 0.001).

Given that VP2 serves as a membrane binding and penetration protein, we performed an alanine substitution of hydrophobic residues at the N-terminus to examine its impact on FCV gRNA release. We successfully obtained viruses with I4A, L5A, I8A, and V11A mutations during virus rescue. However, the VP2 L7A mutation and deletion (aa 4–11) proved lethal to the virus (Fig. 5A). Compared to FCV^WT^, the mutant strains displayed a smaller plaque morphology and lower replication titer (Fig. 5B through D). Viral genome detection results revealed that the gRNA replication of mutant viruses was delayed approximately 1 hour compared to FCV^WT^ (Fig. 5E). We hypothesized that this delayed gRNA replication could be due to a slower rate of RNA release. To test this theory, we measured the RNA release of mutant viruses using the RDL model. In the first 20 min post-virus binding, mutant virus-bound RDLs displayed a lower fluorescence intensity compared to FCV^WT^-bound RDLs, and the time of reaching fluorescence peak in the mutant virus group was longer than the WT virus group (Fig. 5F). This suggests that the RNA release efficiency of mutant viruses is slower than that of FCV^WT^. Therefore, it appears that the alanine substitution of hydrophobic residues at the VP2 N-terminus decreases the RNA release rate in FCV.

## DISCUSSION

**Fig 6:**
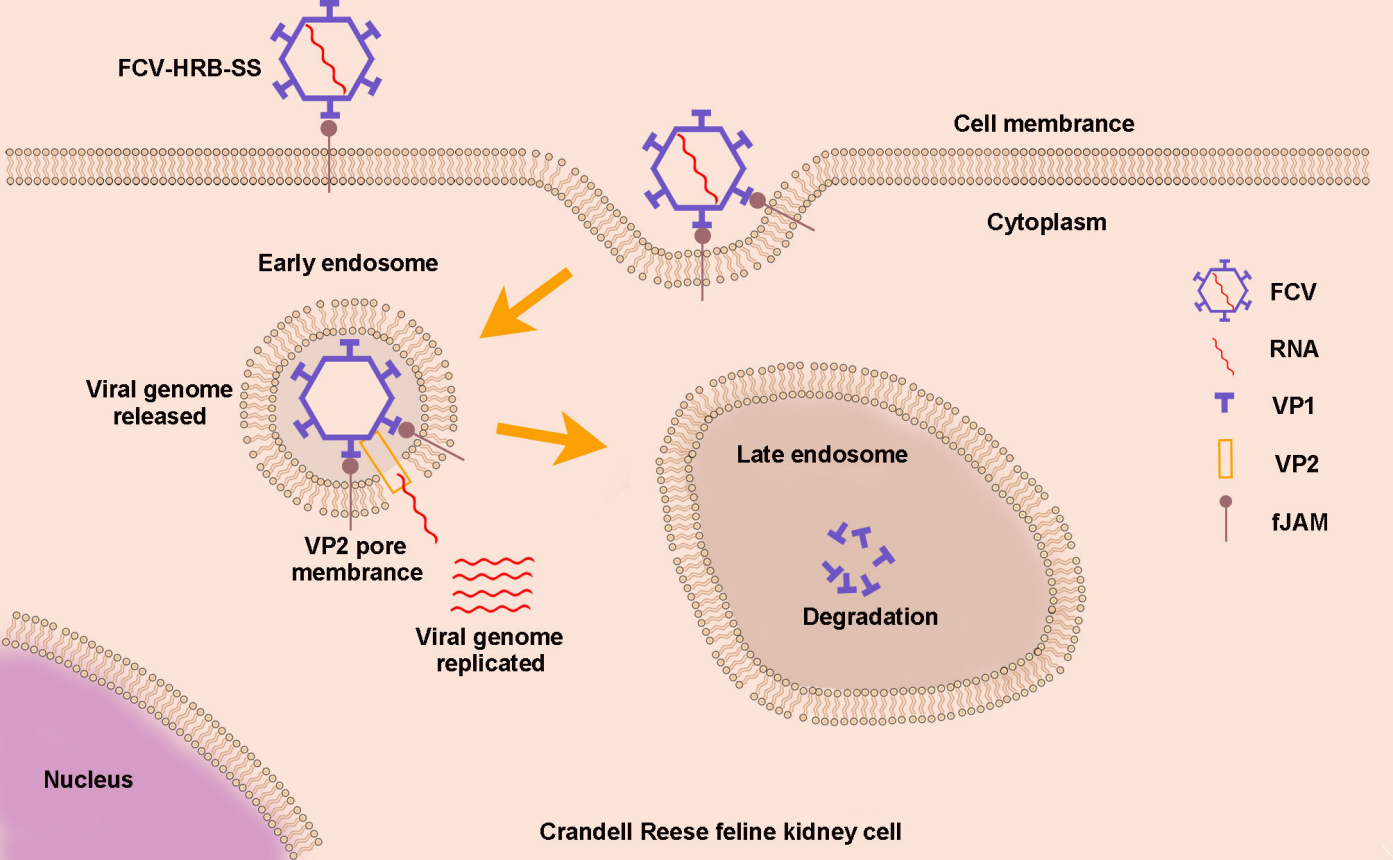
Schematic illustration of VP2 mediates RNA genome release for caliciviruses. FCV attaches to fJAM-A on the host cell surface and enters into early endosomes through clathrin-mediated endocytosis. The gRNA is released into the cytoplasm through pores in the early endosome membrane created by VP2, where it replicates. The viral capsid is degraded in late endosomes.

The initial transfer of viral genetic material into a host cell is crucial for a successful viral infection. However, this process remains unclear for most naked viruses, including caliciviruses, which impede the development of therapeutic drugs targeting this critical stage. In this study, we demonstrate that VP2 mediates the release of FCV RNA by piercing the endosome membrane of infected cells. Based on our findings, we propose a new FCV cell entry model. In this model, FCV initiates infection by first attaching to its cell receptor, fJAM-A, on the host cell surface. It then enters the endosomes via clathrin-dependent endocytosis, releases the gRNA into the cytoplasm through pores in the endosome membranes made by VP2, and begins the genome RNA replication in the cytoplasm. Meanwhile, the capsid proteins degrade in the late endosomes (Fig. 6). This extends our understanding of caliciviruses’ biology.

After binding with receptor, most non-enveloped viruses enter cells through endocytosis (26). The release of the virus genome involves a competitive interaction with the host cell. If this process is too slow, the virus may enter late endosomes, making it susceptible to degradation by the acidic environment and proteases, which can lead to a failed infection (27). Different non-enveloped viruses have adapted various strategies to adjust to the endosome environment and deliver their genome into the cytoplasm at optimum times. For instance, some viruses, such as foot-and-mouth disease virus, release their genome nucleotide acid into the cytoplasm in early endosomes. In contrast, others, such as human rhinovirus type 2 and human astroviruses, travel to late endosomes and rely on the highly acidic environment to successfully deliver their genome (28–31). In our study, we found that FCV releases its gRNA in early endosomes, and the gRNA separates from the capsid rapidly at this stage. We simulated the conditions of early endosomes at pH 6.2 using the RDL model and found that the viral genome is released under these conditions. However, only a few viral particles appeared dark in the negative staining EM images. This suggests that the gRNA-released FCV particles remain compact, which aligns with a previous study showing that empty FCVs exhibit a similar morphology to mature FCV virions under a negatively stained EM examination (32). Furthermore, we observed the gRNA release at pH 7.2; however, it only involved a small portion of the viruses. We also conducted an *in vitro* experiment at a lower pH of 5.2 and found this acid condition led to significant aggregation and breakage of FCV particles (Fig. S3C through E). Therefore, we believe that severe acidic conditions disrupt the regular FCV gRNA release process. These findings enhance our understanding of the cell entry process of caliciviruses.

Debates exist regarding how non-enveloped viruses deliver their genomes across cellular membranes (33). A commonly accepted agreement is that conformational changes in virions during internalization lead to the insertion of hydrophobic viral penetration peptides into a membrane, mediating the release of viral genome nucleotide acid. However, disputes have arisen about the mechanism of how these peptides facilitate viral genome release (23, 33). To date, two membrane disruption manners have been suggested based on the studies of viral penetration peptides from different viruses. These include the formation of small pores (34, 35) and the creation of large-scale fragmentation (36). Given that VP2 is essential to calicivirus reproduction and forms port-like structures after FCV receptor engagement (13, 14), we speculated that VP2 may serve as the membrane penetration peptide to mediate the gRNA release for FCV. Our findings reveal that VP2 can bind to and pore the double-layer membrane of liposomes causing leakage at pH 6.2. The hydrophobic N-terminus of VP2 plays a crucial role in this process. Additionally, mutation of hydrophobic residues in the VP2 N-terminus decreases the gRNA release rate of FCV. These results align with observations made in VP4 of picornaviruses (21, 37). We collectively believe that VP2 serves as the membrane penetration peptide of FCV. It uses its hydrophobic N-terminus to create small pores in the early endosome membrane, mediating the gRNA release.

In summary, our results indicate that FCV releases its gRNA in the early endosomes of infected cells. VP2 serves as the membrane penetration peptide, creating pores in the endosome membranes and delivering the gRNA into the cytoplasm to initiate replication. These findings broaden our understanding of the RNA release mechanism of caliciviruses.

## MATERIALS AND METHODS

### Cells, viruses, and reagents

CRFK cells were maintained at 37°C in 5% CO2 in Dulbecco’s modified Eagle’s medium (DMEM; Gibco) supplemented with 10% fetal bovine serum (FBS; Gibco/Invitrogen), 100 U/mL penicillin, and 100 µg/mL streptomycin (Gibco). The Feline Calicivirus Strain HRB-SS strain (FCV, GenBank accession number KM016908) was used in this study. Amine-reactive dye (Cy5-SE, MedChemExpress) and X-tremeGENE HP DNA Transfection Reagent (Roche) were used to label FCV and transfect plasmid into CRFK cells.

### Virus infection

CRFK cells on coverslips (Nest) were infected with 100 MOI FCV at 4°C for 60 min for attachment. Unattached viruses were washed away with PBS. Pre-warmed post-infection media at 37°C (DMEM with 2% FBS) were added. The cells were then shifted to 37°C to initiate virus entry for a specified duration. Afterward, cells were lysed for RNA and VP1 detection.

### Real-time quantitative PCR

Total RNA was extracted using the RNeasy Mini Kit (Qiagen). This was followed by cDNA synthesis using the PrimeScript RT reagent Kit with gDNA Eraser (Takara). Quantitative PCR was then conducted using the SYBR Green assay (TaKaRa) on a QuantStudio 3 instrument (Applied Biosystems). The primers, designed with SnapGene software (version 5.3 software), targeted the ORF3 of FCV. The sequences were forward: 5′-CCAATACAAT TGGGAAAGCTC-3′; reverse: 5′-CCTCTGCTCAAGAATCTTGT-3′. Results were normalized by β-Actin mRNA expression, using the primers: forward:5′-CAGGTGATCACCATCAGGCAACG-3′; reverse:5′-GACAGCACCGTGTTAGCGTAGAG GT-3′.

### Western blotting analysis

CRFK cells were lysed with 100 µL of reducing SDS sample buffer and heated at 100°C for 10 min. Twenty microliters of the supernatant was loaded onto a 12% SDS polyacrylamide gel and semi-dry transferred onto a PVDF membrane. The membrane was blocked with 5% milk powder in PBS and incubated with either a mouse anti-VP1 antibody (Abcam) or a mouse anti-GAPDH antibody (GeneTex) for 1 h. After washing the membrane, the bound antibodies were detected using an Alexa Fluor 488 labeled goat anti-mouse IgG (Thermo Fisher, diluted 1:5,000). VP1 expression levels were quantified by analyzing the VP1 bands with ImageJ software. The data were normalized by the respective control GAPDH bands and correlated with the quantity at 0 min.

### Virus purification and label with Cy5

Cell cultures containing FCV were collected when 70%–80% of the cells showed a cytopathic effect and then centrifugated at 700 *g* for 15 min to remove cellular debris. This was followed by centrifugation at 120,000 *g* at 4°C to pelletize the virus particles (38). Crude virus particles were resuspended in PBS, loaded onto a 15%–54% iodixanol gradient in PBS, and centrifuged at 350,000 *g* for 3 h at 4°C. Purified virus particles were extracted from the gradient, concentrated by centrifugating at 120,000 *g* for 2 h, resuspended in 100 µL of PBS. To label the FCV capsid, purified viruses were incubated with the Cy5-SE in a carbonate buffer (pH 9.3) at room temperature with gentle agitation for 1 h. Unbound dye was removed by performing three high-speed centrifugations at 120,000 *g* for 2 h at 4°C. Virus titers were determined using the median tissue culture infective dose (TCID_50_) in CRFK.

### Transfection and Cy5-labeled FCV infection

1 × 10^5^ CRFK cells on coverslips were transfected with 1 µg of eGFP-rab5 or eGFP-rab7 plasmid for 24 h prior to infection with 100 MOI Cy5-labeled FCV. After virus entry for a specified duration, cells were fixed by 4% paraformaldehyde for 10 min and permeabilized by 70% ethanol overnight at 4°C.

### Single-molecule fluorescent *in situ* hybridization methodology

The smiFISH protocol and probe design were following the procedure outlined in reference (39). Briefly, 81 primary probes were designed. Each of them consists of two parts: a specific sequence complementary to the genome sequence of FCV HRB-SS, and a 28-nt long readout sequence that is identical among all primary probes (FLAP sequence). Additionally, two Cy-3-labeled fluorescent probes, which are complementary to the FLAP, were synthesized and pre-annealed to the primary probes, forming Flap-structured duplexes. The fixed and permeabilized CRFK cells were rinsed with PBS and then incubated in 15% formamide in 1× SSC buffer for 15 min. Next, a 50-µL aliquot of the hybridization mixture (15% formamide, 1 × SSC, 10.6% dextran sulfate, 2 mM vanadylribonucleoside complex, 20 µg bovine serum albumin, 2 µL Flap-structured duplexes, and 340 mg *E. coli* tRNA per mL) was applied to a 10-cm Petri dish. The coverslip was gently placed on the droplet, with cells facing downward, and hybridized at 37°C overnight. After two 30 min washes with 15% formamide in 1 × SSC, the slides were mounted in Fluoroshield with DAPI (Sigma). Observation was made using an LSM800 confocal microscope (Zeiss) with a 63×/1.4 oil objective. Images were acquired using a Zeiss Zen Blue 2.3 Lite system equipped with an Airyscan module.

### Image analysis

The confocal microscope images were analyzed using Arivis Vision4D software (arivis) (40). Colocalized events between the RNA and capsid were identified if the distance between the centers of the nearest red and green fluorescence spots was less than 0.14 µm (3.96 pixels). More than 1,000 fluorescence spots were quantified. The borders of cells or early and late endosomes were also defined by Arivis to further analyze the fluorescence signal within them.

### Transmission electron microscopy

CRFK cells were infected with 1,000 MOI FCV for 10 min and then fixed with 2.5% glutaraldehyde for 16 h. The fixed cells were dehydrated through a graded ethanol series (50%, 70%, 90%, 95%, 99%, and 100%) at 4℃. The cells were embedded in 812 Epon resin and polymerized at 70℃ for 2 days. Ultrathin sections were prepared using an UC6 ultra-microtome (Leica Microsystems) and stained with 1% uranyl acetate and 1% lead citrate. The sections were examined with an H-7650 transmission electron microscope (Hitachi, Tokyo, Japan) operating at 80 kV.

### Feline JAM-A expression and purification

The gene encoding the extracellular domain of Feline JAM-A (fJAM-A) (aa 21–232) was synthesized with a 6 × HIS tag fused at its C-terminus (BGI). It was then inserted into the pGEX-6P-1 vector (GE Healthcare) with a GST tag at its N-terminus. For fJAM-A-His expression in *E. coli*, the pGEX-6P-1-fJAM-A-His plasmid was transfected into BL21 (DE3) cells and induced with 0.1 mM isopropyl β-d-1-thiogalactopyranoside (IPTG) at 16°C for 16 h. The cells were then harvested, resuspended in PBS, and lysed by sonication. After affinity purification and GST tag removal, fJAM-A-HIS was further purified using a Resource Q column (GE Healthcare) and size-exclusion chromatography with a Superdex 200 column (GE Healthcare) in HEPES buffer (50 mM HEPES, pH 7.2, 50 mM NaCl).

### Formation of receptor-decorated liposomes

Liposomes were prepared with phosphatidylethanolamine, phosphatidylcholine, sphingomyelin, cholesterol, and phosphatidic acid (Avanti Polar Lipids) dissolved in chloroform at a molar ratio of 1:1:1:1.5:0.3. Nickel-charged liposomes, employed for His-tagged receptor binding, were formed by incorporating DGS-NTA-Ni [*1,2-dioleoyl-sn-glycero-3-((N-(5-amino-1-carboxypentyl) iminodiacetic acid) succinyl*); Avanti Polar Lipids] at a final concentration of 10% (wt/wt) (18). To visualize liposome in the flotation assay, we added rhodamine B-labeled PE (Avanti Polar Lipids) into the solution at a final concentration of 0.5% (wt/wt). The mixture was evaporated using an argon gas stream to form a lipid film and then desiccated under vacuum overnight. The lipid film was hydrated in HEPES buffer and extruded through a 0.1-µm pore size membrane (Avanti Polar Lipids) to generate homogeneous liposomal populations. At room temperature, 0.43 mg/mL of fJAM-A-His was incubated with 1 mg/mL nickel-charged liposomes in a 1:10 ratio (vol/vol) for 30 min. The formation of RDLs was assessed using a flotation assay and immunoelectron microscopy. To assess the viral binding capacity of the RDL, 1 mg/mL RDLs were mixed with 1 mg/mL purified FCV at a ratio of 20:1 (vol/vol) at room temperature for 30 min. The mixture was then analyzed using a flotation assay, TEM, and cryo-EM.

### Liposome flotation assay

The assay was conducted by following the previously described procedures (34). The RDL mixture was adjusted to a sucrose concentration of 1.4 M, loaded at the bottom of a sucrose gradient with two layers (0.8 M and 0.5 M sucrose from bottom to top), and then centrifuged at 350,000 *g*. for 3 h at 4°C, resulting in four distinct layers. Liposomes were identified with rhodamine, while receptors and viruses were analyzed through WB using anti-His antibody (GeneTex) and anti-VP1 antibody, respectively.

### Immunoelectron microscopy

RDL were adsorbed onto freshly glow-discharged carbon grids, blocked with 3% Bovine Serum Albumin for 30 min, and then incubated with polyclonal antibodies against fJAM-A (mouse) and a goat anti-mouse antibody conjugated to 5-nanometer gold particles (sigma). Finally, the grids were negatively stained and then analyzed using a TEM.

### RNA release assay

A dried nickel-chelating lipid film was rehydrated in a buffer containing 50 mM HEPES (pH 7.2), 50 mM NaCl, YoPro-1 (ThermoFisher) at a 1:500 ratio (vol/vol), and 1% glucose. RDLs with a lipid concentration of 1 mg/mL were prepared. RNase A (Sigma) was added to achieve a final concentration of 200 µg/mL to eliminate the background signals outside the liposomes. Then, 10 µL of 1 mg/mL virus was incubated with 90 µL of RDLs. The RNA release assay measured the fluorescence signal from YoPro-1 binding with FCV RNA releasing into RDLs. To assess the pH impact on RNA release, the pH was adjusted using 1 M sodium acetate (pH 3.0) and verified with pH paper. Fluorescence measurements were taken every 5 min for 1 h at 37°C using a PHERAstar FS plate reader (BMG Labtech) with excitation and emission wavelengths of 490 nm and 520 nm, respectively. For visualizing the RNA release, the RDL and virus were incubated at different pH conditions for 30 min at 37°C and imaged using an inverted fluorescence microscope (EVOS FL, Life, USA) with a 20 × objective.

### Negative-stain electron microscopy

The prepared nickel-charged liposomes or RDL-FCV mixture were applied to freshly glow-discharged carbon grids. These grids were stained with 2% uranyl acetate and examined under a Hitachi H-7650 transmission electron microscope (Hitachi High Technologies, Tokyo, Japan), operated at an acceleration voltage of 80 kV.

### Cryo-electron microscopy

To prepare cryo-EM samples of RDL-FCV complex and the mixture of VP2-GST and liposomes, a 3.5 µL droplet of sample was loaded onto a glow-discharged Quantifoil grid (Quantifoil, R 1.2/1.3, 200-mesh, Au). Excess liquid was removed from the grid surface before it was rapidly plunged into liquid ethane. The grids were transferred into a Titan G4 electron microscope operating at an acceleration voltage of 300 kV. Micrographs were acquired on a K2 Summit camera (Gatan, Pleasanton, California) in super-resolution mode with an effective magnification of 13,000 × using SerialEM28.

### Cloning and purification of recombinant VP2-GST and mutant VP2 proteins

The VP2 cDNA fragment was synthesized using RT-PCR with template RNA extracted from the FCV particles by the following RT-PCR primers: sense primer 5′-TACATATGAACTCAATATTGGGACT-3′ and antisense primer 5′-ATAACCTAGTATAGGGGACATATTCTTAAACAGGT-3′. The cDNA fragment of VP2-GST^Δ4-11^ was synthesized using the same antisense primer and a sense primer 5′-TTAAGAAGGAGATA TACATATGAACTCAATATTGGGACT-3′. The cDNA fragments of VP2-GST with single amino acid mutations were amplified with the same antisense primer as for amplifying VP2-GST, and the sense primers were introduced corresponding base to the position of mutation. GST was amplified by PCR using primers GST-F (5′-ACCTGTTTAAGAA TATGTCCCCTATACTAGGTTA-3′) and GST-R (5′-CTTGTCGACGGAGCTCGAATTCTTATT TTGGAGGATGG-TCGCC-3′) from pGEX 6 P-1 (Invitrogen). Overlapping templates were created from these PCR products by identifying complementary primer sequences (denoted by underlined regions). These were used in a subsequent PCR to produce a cDNA encoding VP2-GST and mutant VP2-GST. To synthesize the GST^N11-VP2^ cDNA fragment, we used the antisense primer GST-R and GST^N11-VP2^-F (5′-TTAAGAAGGAGATATACATATGAACTCAATATTGGGACTTATTGATACTG TCTCCCCTATACTAGGTTATTGGAAAATTA-3′). The cDNA fragments above were cloned into the pET29a plasmid, and the recombinant plasmids were transformed into *E. coli* BL21(DE3) Chaprone. Protein production was induced with 0.2 mM IPTG at 15°C for 16 h. The bacterial culture was harvested, resuspended in tris buffer (25 mM Tris, pH 8.0, 50 mM NaCl, and 3 mM DTT), and lysed via sonication. The recombinant proteins were purified using a GSTrap FastFlow column (GE Healthcare) and gel filtration size-exclusion chromatography (Superdex 200, GE Healthcare) in HEPES buffer.

### Membrane binding assay

One milligram per milliliter fluorescently labeled liposomes (∼1.5 mM) was co-incubated with 4 µM VP2-GST, VP2-GST^Δ4-11^, or GST at 37°C for 30 min under varying pH conditions. Subsequently, a liposome flotation assay was performed and fractions were analyzed by WB.

### Leakage assays

Liposomal leakage was measured using the ANTS-DPX assay method (41). Liposomes (1 mg/mL) were formulated with 25 mM 8-aminonaphthalene-1,3,6-trisulfonic acid (ANTS) and 90 mM quencher 2,4-dinitrophenyl (DPX). Any unencapsulated dye molecules were removed using a HiTrap desalting column. The dilution of ANTS-DPX in the surrounding medium due to liposomal leakage resulted in increased ANTS fluorescence because of reduced DPX quenching. After the target protein was added, ANTS fluorescence was measured at 30 s intervals (excitation at 355 nm and emission at 520 nm) using a Fluoromax spectrometer at 37°C. The equilibrium point in fluorescence intensity was determined. Then, 1% Triton X-100 was added to induce complete vesicle lysis, representing 100% leakage.

The leakage was quantified with the following equation:


$$Percent\;leakage=((F-F_{0})/(F_{100}-F_{0}))\times 100$$

Here, “*F*” represents the fluorescence intensity after protein addition, “*F*_0_” is the fluorescence of the intact vesicles before protein addition, and “*F*_100_” corresponds to the fluorescence value after 1% Triton X-100 introduction (41).

### Construction of infectious cDNA clone and virus rescue

To establish a reverse genetic platform for FCV, the full-length genome of FCV HRB-SS was chemically synthesized. It was appended with a hepatitis D virus ribozyme (HdvRz) sequence 3′ end and inserted into the low-copy vector pOK12-CMV, creating the recombinant plasmid pFCV-HRB-SS. The plasmid was transfected into CRFK cells for virus rescue (42). After three passages in CRFK cells, the rescued viruses were harvested by freezing and thawing. To introduce Alanine substitutions into the VP2 sequence at specific positions, the modified DNA fragment was amplified from the pFCV-HRB-SS plasmid using site-directed mutagenesis PCR, which used the primers to introduce the corresponding base to the mutation site. This altered fragment was then inserted into the correct sites of the pFCV-HRB-SS plasmid and transfected into CRFK for virus rescue.

### Plaque assay

A total of 100 TCID^50^ virus particles were added to CRFK monolayer in a 6-well plate and incubated for 60 min. Following three PBS washes, growth medium containing 0.6% agarose overlay was applied. Approximately 48 h later, the agarose overlay was removed. The cells were then stained with crystal violet and subsequently imaged. The plaque area was analyzed using Image J (version 1.8.0). To conduct the RNA protection assay, FCV (40–60 pfu) were added to monolayer CRFK cells together with RNase A at concentrations of 0, 0.01, 0.1, or 1 mg/mL and incubated for 60 min at 37°C. Then, the RNase A-containing inoculum was replaced with 0.6% agarose containing medium. After 48 h incubation, cells were fixed with 4% paraformaldehyde and stained with crystal violet. The relative plaque-forming units (pfu 1 m1^−1^) were standardized by the percentage of no RNase A control.

### One-step growth curve assay

The assay was performed as described previously (43). In brief, the virus (10 MOI) was adsorbed to CRFK monolayers for 1 h (time zero) and then supplemented with CRFK growth medium. Samples were taken at various times post-infection, frozen, and stored at −80°C for later titration. The virus titer at each time point was expressed as log_10_ TCID_50_ and standardized by subtracting the log_10_ (TCID_50_ 100 μ1^−1^) at time zero. The means and standard deviations (SD) from three independent experiments are presented.

### Statistical analysis

Data were analyzed with GraphPad Prism software (version 9.00). The independent-sample *t*-test was used for comparisons among two groups, and one-way analysis of variance (ANOVA) for comparisons among three or more groups. The results are presented as means ± SD. Asterisks indicate statistical significance (*P < 0.05, **P < 0.01, ***P < 0.001).

## Data Availability

The data that support the findings of this study are available from the corresponding author upon reasonable request.

## SUPPLEMENTAL MATERIAL

Fig. S1 jvi.00350-24-s0001.tif Preparation and characterization of Cy5-labeled FCV.

Fig. S2 jvi.00350-24-s0002.tif Characterization of Cy3-labeled probes for detecting FCV RNA in infected cells.

Fig. S3 jvi.00350-24-s0003.tif Detection of FCV RNA release at pH 5.2 condition.

Fig. S4 jvi.00350-24-s0004.tif Preparation of mutant VP2 proteins.

Supplemental figure legends jvi.00350-24-s0005.docx Legends for Fig. S1 to S4.

ASM does not own the copyrights to Supplemental Material that may be linked to, or accessed through, an article. The authors have granted ASM a non-exclusive, world-wide license to publish the Supplemental Material files. Please contact the corresponding author directly for reuse.
