# Supplementary figures and images for: VP2 mediates the release of the feline calicivirus RNA genome by puncturing the endosome membrane of infected cells
Source: J Virol. 2024 Apr 9;98(5):e00350-24. doi: 10.1128/jvi.00350-24 (PMC11092339; doi:10.1128/jvi.00350-24)

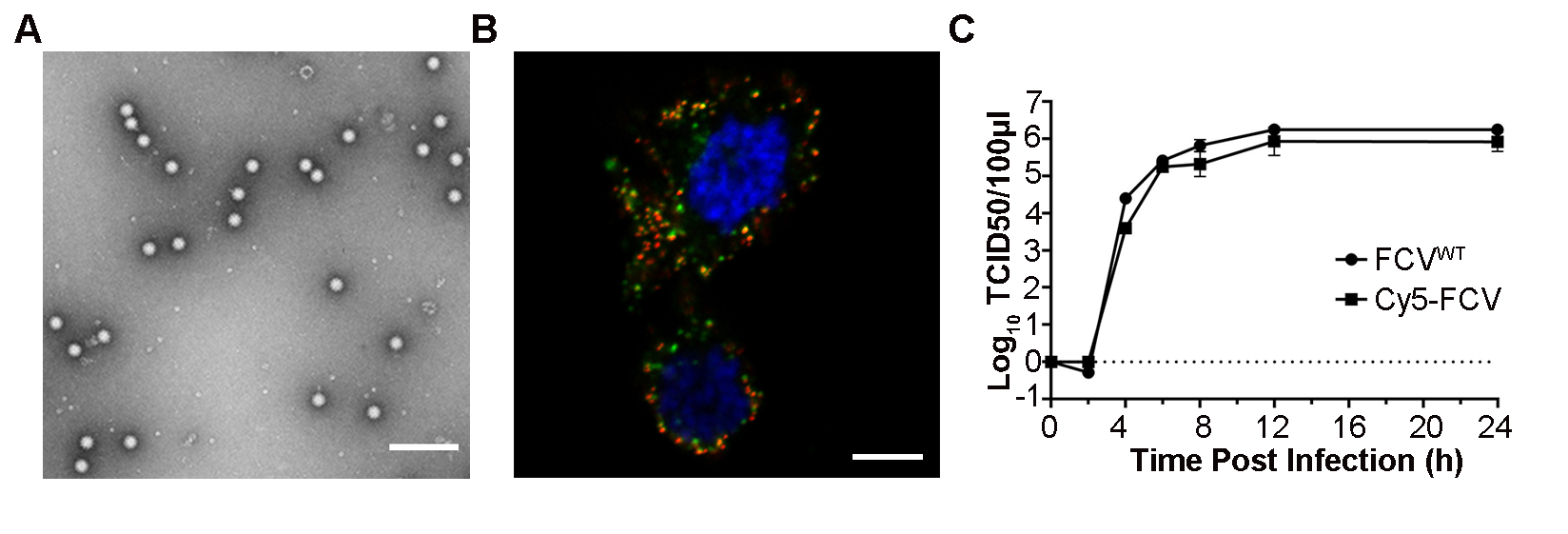

Supplement: Fig. S1 — Preparation and characterization of Cy5-labeled FCV. [file jvi.00350-24-s0001.tif]

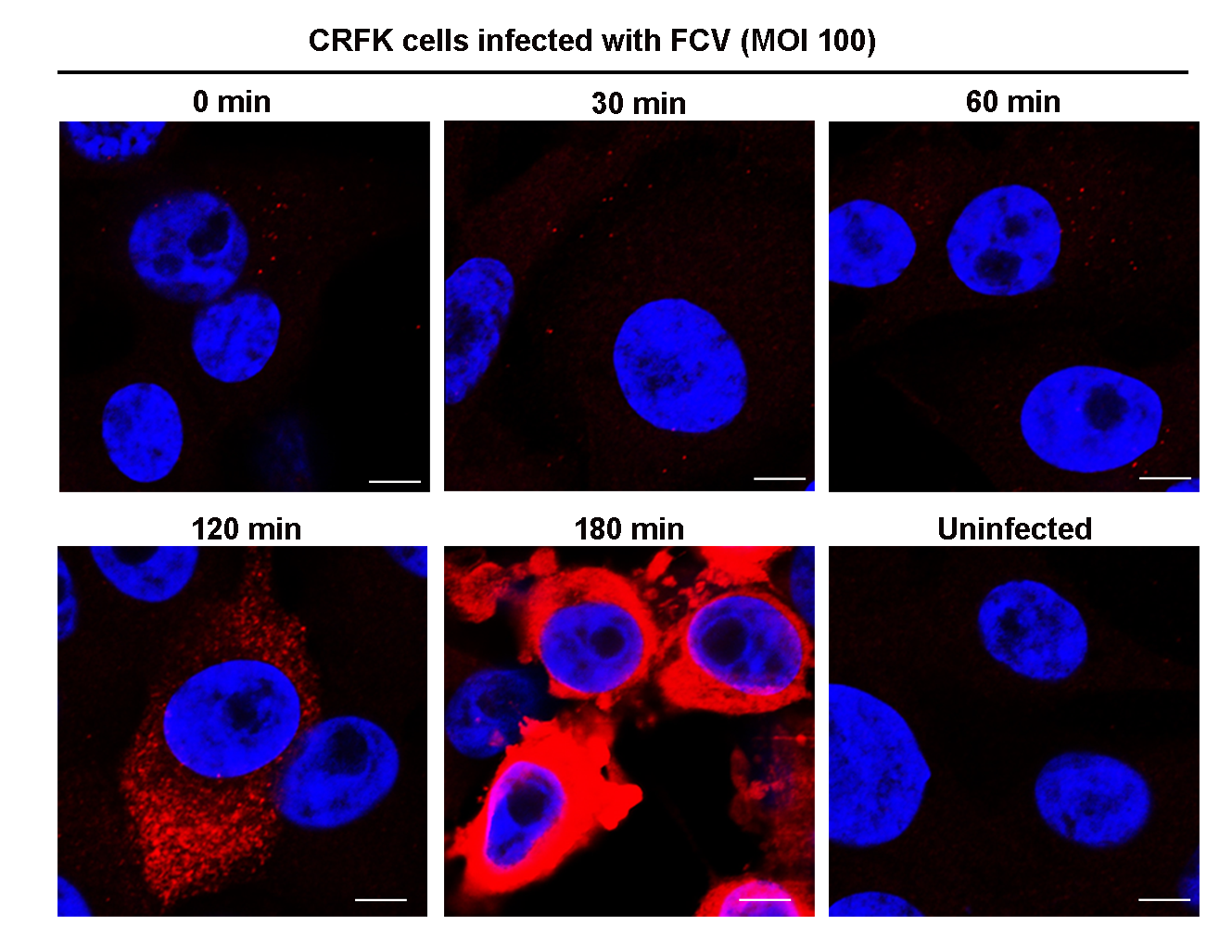

Supplement: Fig. S2 — Characterization of Cy3-labeled probes for detecting FCV RNA in infected cells. [file jvi.00350-24-s0002.tif]

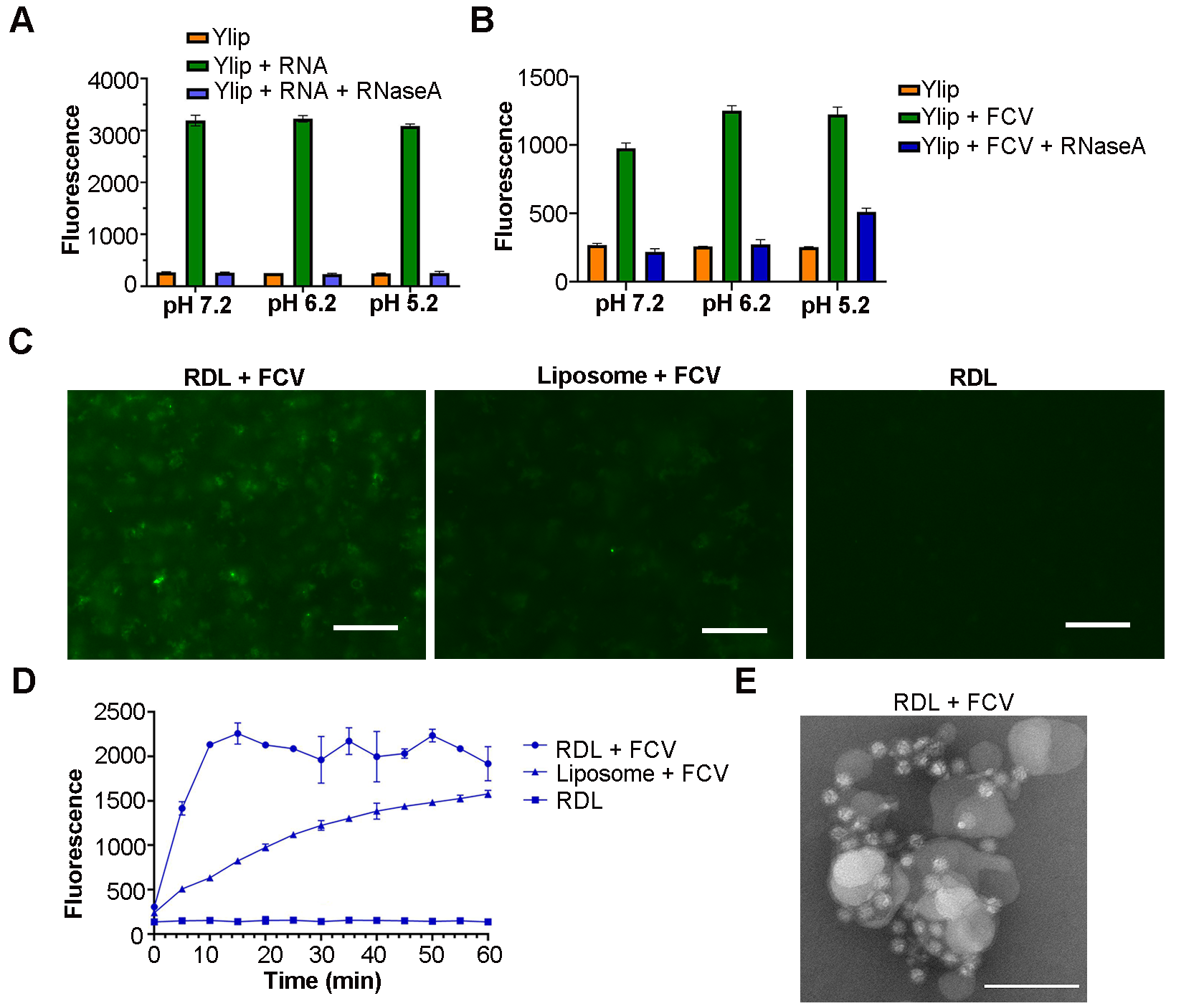

Supplement: Fig. S3 — Detection of FCV RNA release at pH 5.2 condition. [file jvi.00350-24-s0003.tif]

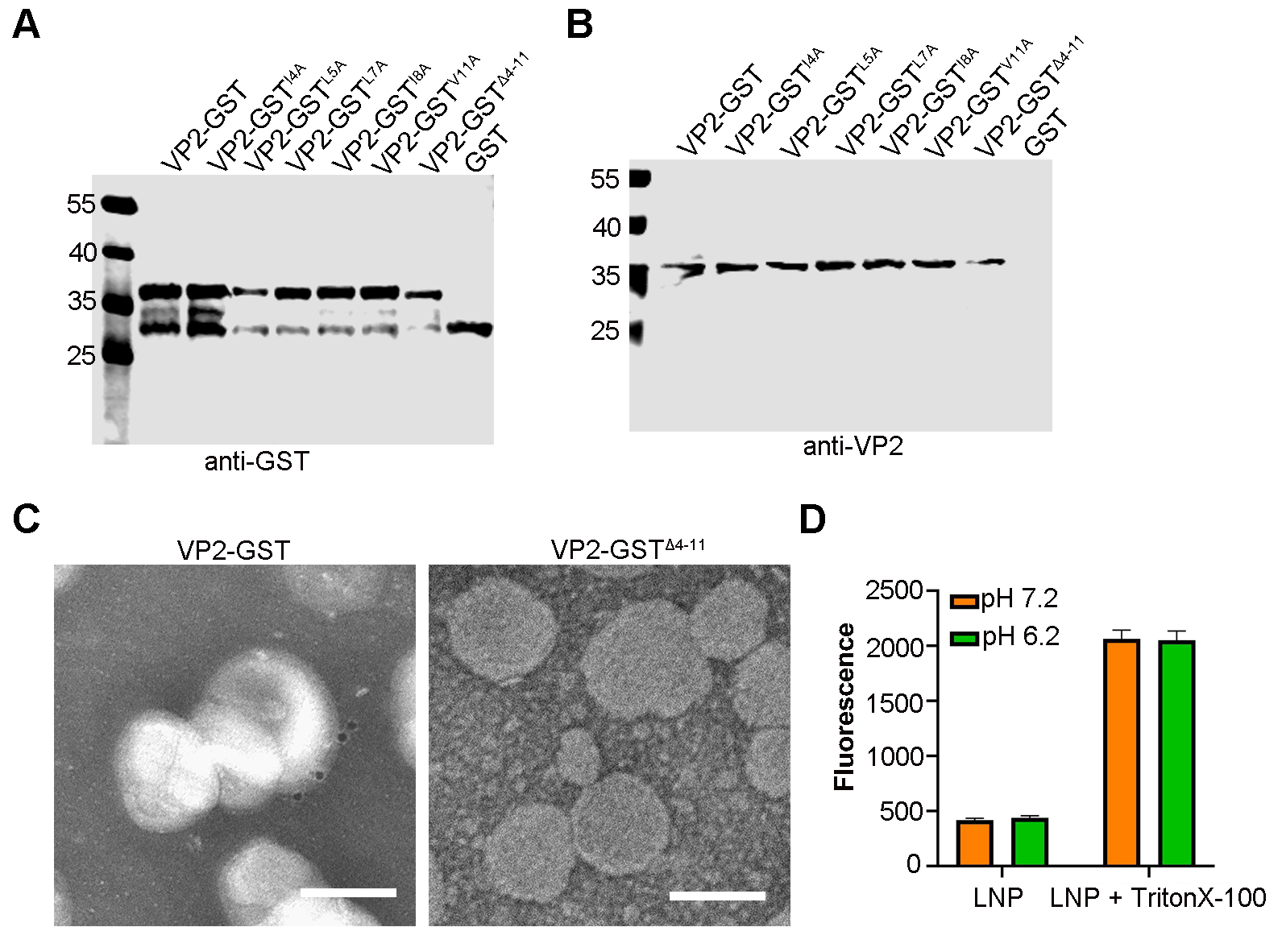

Supplement: Fig. S4 — Preparation of mutant VP2 proteins. [file jvi.00350-24-s0004.tif]
