# Supplementary material for: VP2 mediates the release of the feline calicivirus RNA genome by puncturing the endosome membrane of infected cells
Source: J Virol. 2024 Apr 9;98(5):e00350-24. doi: 10.1128/jvi.00350-24 (PMC11092339; doi:10.1128/jvi.00350-24)
Supplement: Supplemental figure legends — Legends for Fig. S1 to S4. [file jvi.00350-24-s0005.docx]

**Fig S1** **Preparation and characterization of Cy5-labeled FCV.** (A) TEM image of the purified Cy5-labeled FCV. Scale bar = 200 nm. (B) Indirect immunofluorescence image showing the attachment of Cy5-labeled FCV with CRFK cells. The nucleus was stained with DAPI. Scale bar is 5 μm. (C) One step growth curve of Cy5-labeled FCV compared to FCV^WT^. Error bars represent SD from three independent experiments.

**Fig S2 Characterization of Cy3-labeled probes for detecting FCV RNA in infected cells.** Confocal microscope images show the different RNA levels detected by smiFISH at indicated time post infection. CRFK cells were infected with FCV (100 MOI) for indicated time and then fixed to perform smiFISH. The nucleus was stained using Fluoroshield^TM^ with DAPI. Scale bar indicates 5 μm.

**Fig S3 Detection of FCV RNA release at pH 5.2 condition.** Diminishing effects of contaminant signals from RNA (A) and FCV (B) outside the liposome with RNase A. Ylip: refers to liposomes with a solution that contains Yopro-1, as shown in Figure 3A. Under different pH conditions, 800 ng FCV RNA or 10 μg purified FCV was added. The RNase A was added at a final concentration of 200 μg/ml. The fluorescence signal was recorded after 5 min incubation. (C) Fluorescence microscopy images display the fluorescent signal from FCV-bound RDL at pH 5.2 for 30 min. Bar, 100 μm. (D) Line plot shows the change in the fluorescence intensity of the mixtures at pH 5.2. Error bars show the SD from three independent experiments. (E) TEM image shows FCV particles attached to the surface of RDL at pH 5.2. Bar, 200 nm.

**Fig S4 Preparation of mutant VP2 proteins**. (A and B) Purified VP2-GST and its mutant proteins were verified using WB with an anti-GST antibody (ThermoFisher) (A) and anti-VP2 antiserum (B). (C) Immunoelectron microscopy images show the attachment of VP2-GST on the liposomes at pH 6.2. Scale bar, 100 nm. (D) Bar chart shows the leakage of liposome induced by adding 1% TritionX-100 at different pH conditions. LNP: liposomes containing ANTS-DPX.
